# Cortical bone continuum damage mechanics constitutive model with stress triaxiality criterion to predict fracture initiation and pattern

**DOI:** 10.3389/fbioe.2022.1022506

**Published:** 2022-10-17

**Authors:** D. S Cronin, B Watson, F Khor, D Gierczycka, S Malcolm

**Affiliations:** ^1^ Department of MME, University of Waterloo, Waterloo, ON, Canada; ^2^ Honda Development and Manufacturing of America, Raymond, OH, United States

**Keywords:** cortical bone, constitutive model, continuum damage mechanics, finite element model, femur, bone fracture

## Abstract

A primary objective of finite element human body models (HBMs) is to predict response and injury risk in impact scenarios, including cortical bone fracture initiation, fracture pattern, and the potential to simulate post-fracture injury to underlying soft tissues. Current HBMs have been challenged to predict the onset of failure and bone fracture patterns owing to the use of simplified failure criteria. In the present study, a continuum damage mechanics (CDM) model, incorporating observed mechanical response (orthotropy, asymmetry, damage), was coupled to a novel phenomenological effective strain fracture criterion based on stress triaxiality and investigated to predict cortical bone response under different modes of loading. Three loading cases were assessed: a coupon level notched shear test, whole bone femur three-point bending, and whole bone femur axial torsion. The proposed material model and fracture criterion were able to predict both the fracture initiation and location, and the fracture pattern for whole bone and specimen level tests, within the variability of the reported experiments. There was a dependence of fracture threshold on finite element mesh size, where higher mesh density produced similar but more refined fracture patterns compared to coarser meshes. Importantly, the model was functional, accurate, and numerically stable even for relatively coarse mesh sizes used in contemporary HBMs. The proposed model and novel fracture criterion enable prediction of fracture initiation and resulting fracture pattern in cortical bone such that post-fracture response can be investigated in HBMs.

## 1 Introduction

The modeling of hard tissue response under load, fracture initiation, and post-fracture response for different modes of loading is critical for the prediction of Crash Induced Injuries in advanced finite element (FE) Human Body Models (HBMs) (Schmitt et al., 2019). Cortical bone is present in the diaphyses of long bones, as a thin shell in the epiphyses of long bones and surrounding the flat bones (Shore et al., 2013), where fractures involving cortical bone are important in injury biomechanics and may be associated with serious or greater injury levels (i.e., Abbreviated Injury Scale AIS 3+) (AAAM, 2015). Furthermore, predicting fracture pattern and post-fracture response of hard tissue is critical for the future possibility of assessing injury risk to underlying soft tissues. Current HBMs often model cortical bone using an isotropic, elastic-plastic material model incorporating a yield surface to predict the response of hard tissue (Yang, 2017), while failure is modeled using element erosion at a specified effective plastic strain (DeWit, & Cronin, 2012). Two benefits of this simplified approach are computational efficiency and numerical robustness, ensuring the calculation runs to completion with the coarse mesh sizes (e.g. 1–3 mm) used in contemporary HBMs. Such constitutive models can predict the onset of failure arising from uniaxial tension loading, since the material parameters and failure criterion are usually calibrated to this load case, but are limited in predicting failure initiation in other modes of loading (Khor et al., 2018), and have not been successful at predicting bone fracture pattern. Although detailed microstructural models have been proposed for cortical bone (Dapaah et al., 2020), such models with elements on the order of 10 microns in the region of interest, are too computationally expensive for whole body HBM simulations.

**FIGURE 1:**
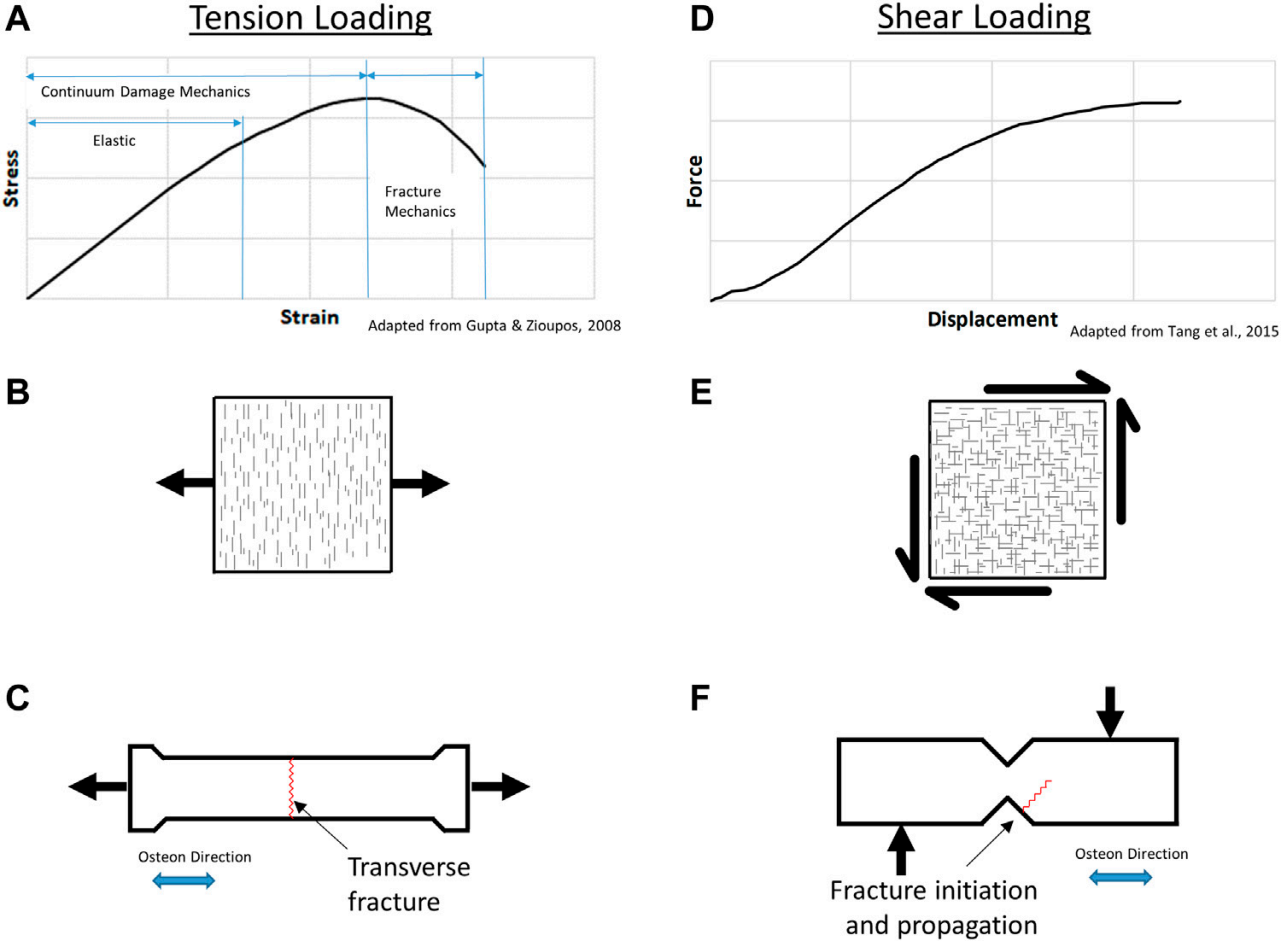
Cortical bone tension response **(A)**, microcrack damage **(B)**, and resulting transverse fracture **(C)**; shear response **(D)**, microcrack damage **(E)**, and resulting fracture **(F)**.

Current state-of-the-art HBMs require a constitutive model, which is functional for coarse element sizes (∼1–3 mm) used in HBMs, relative to contemporary micro-models. Previous research (Khor et al., 2018) has demonstrated the need for asymmetric tension-compression behavior and orthotropy. Cortical bone also exhibits damage or post-yield behavior and complex fracture patterns depending on the mode of loading (Figure 1). In the present study a continuum damage mechanics (CDM) material model with material direction-dependent damage was integrated with a proposed stress triaxiality-based fracture criterion to predict the onset of failure and resulting bone fracture pattern. The fracture criterion parameters were fit to experimental data. The resulting predicted fracture patterns were assessed using three experimental load cases.

### 1.1 Cortical bone structure, mechanical properties and constitutive models

Approximately 80% of the human skeletal bone mass is cortical bone (Cowin, 2001) comprising inorganic hydroxyapatite, organic Type I collagen phases, other collagen types, non-collagenous proteins, and water (Keaveny et al., 2003). The stiffness of bone has been associated with the inorganic phase (hydroxyapatite) (Saito, & Marumo, 2015); while the organic content (primarily Type I collagen) is associated with the tensile properties and post-yield deformation of the bone. The structure of cortical bone is hierarchical in nature, beginning with the inorganic and organic phases (∼0.1 micron), increasing to lamellae (∼1–10 micron) and cylindrically-shaped osteons (200 µm diameter, 1–3 mm length). Within the long bones of the body, the osteons are oriented along the diaphyseal or long axis of the bone (Idkaidek & Jasiuk, 2017). Cortical bone has a higher stiffness and strength along the longitudinal or osteon direction than the transverse direction, with orthotropic shear properties (Tang et al., 2015). In addition, the properties of cortical bone may vary with age, sex and lifestyle (Bruno et al., 2014), and the measured mechanical properties may vary with hydration, embalming (Sanborn et al., 2016; Öhman et al., 2008; Stefan et al., 2010) and specimen manufacturing method (Cowin, 2001). A previously reported set of mechanical properties was presented by Khor et al. (2018) (Table 1).

**TABLE 1:** Cortical bone material property summary (Khor et al., 2018).

Material Parameter	Value
Longitudinal Young’s modulus (E_1_) (Tension/Compression)	16.4 GPa / 17.28 GPa Reilly et al. (1974)
Transverse Young’s modulus (E_2_=E_3_)	12.7 GPa Reilly and Burstein (1975)
Longitudinal Ultimate Strength (S_1_)	135 MPa Reilly et al. (1974)
Transverse Ultimate Strength (S_2_, S_2_)	53 MPa Reilly et al. (1974)
Shear Strength (S_12_,S_23_, S_31_)	68 MPa Reilly et al. (1974)
Shear Modulus (G_23_=G_32_)	5.1 GPa Tang et al. (2015)
Shear Modulus (G_13_=G_12_=G_32_=G_21_)	3.9 GPa Tang et al. (2015)
Bulk Modulus (K)	10.31 GPa
Poisson’s ratio (ν_12_=ν_13_)	0.235 Ashman et al. (1984)
Poisson’s ratio (ν_32_)	0.376 Ashman et al. (1984)
Density	2000 kg/m^3^

Under uniaxial tension loading, cortical bone material response is characterized in three phases (Figure 1A): an initial elastic response; continuum damage mechanics response, where damage in the material accumulates as microcracks oriented perpendicular to the applied load direction or principal stress (Figure 1B); and the fracture mechanics region where damage localizes leading to the initiation and propagation of a fracture (Gupta & Zioupos 2008). The microcracks occur perpendicular to the applied stress, providing energy absorption prior to failure, where the resulting crack paths typically follow the osteon cement lines (Zhai et al., 2019a). Experimental studies have reported sublaminar microcracking in bone but were focused on compressive loading (Ebacher et al., 2012), whereas the fracture of cortical bone has been reported to initiate in regions subjected to tensile loading (Zhai et al., 2019b; Wolfram & Schwiedrzik, 2016). Tension loading parallel to the osteons at the coupon level generally leads to transverse fracture of the specimen, perpendicular to the applied load (Figure 1C). The strain dependence of cortical bone failure has been reported (Nalla et al., 2003), owing to diffuse microcracking damage that may be pressure-dependent.

Several shear tests have been applied to test bone including the rail shear test, the torsion tube, and cross-beam specimen, with the limitation that some methods inadvertently generate tension in the test specimen (Cowin, 2001). Damage reported from shear loading (Figure 1D) occurs as microcracks in orthogonal directions (Figure 1E); however, failure is not reported to occur in the zone of maximum shear within the notched area or ligament of the test specimen. The Iosipescu test involves shear loading using rigid clamping fixtures to induce uniform shear in the ligament of the test specimen (Tang et al., 2015). Shear loading using the notched shear (Iosipescu) geometry results in a non-intuitive fracture initiation away from the notch and propagating at an angle into the sample (Figure 1F) (Tang et al., 2015), demonstrating the complex behavior of cortical bone.

Cortical bone constitutive models have been investigated at the micro-scale (Idkaidek & Jasiuk, 2017; Abdel-Wahab et al., 2012; Ascenzi et al., 2013; Demirtas et al., 2016; Ural & Vashishth, 2006; Ural et al., 2011; Feerick et al., 2013), at the macro-scale at coupon level (Garcia et al., 2009; Li et al., 2013), and at the scale of whole bones (San Antonio et al., 2012; Ariza et al., 2015; Asgharpour et al., 2014; Keyak et al., 1997; Iwamoto et al., 2005; Schileo et al., 2008; Zysset et al., 2013). Contemporary HBMs often use linear isotropic and symmetric metals plasticity constitutive models (DeWit, & Cronin, 2012; Asgharpour et al., 2014; Untaroiu et al., 2013). A recent study (Khor et al., 2018) identified the importance of including asymmetry and orthotropy to predict the response and failure of whole bones. In general, orthotropic material properties were essential to predict the onset of failure for whole bone simulations in two primary modes of loading (bending and shear); however, the predicted fracture patterns were not in agreement with reported patterns when using plasticity-based metal constitutive models. Incorporation of tension-compression asymmetry generally improved the gross kinetics and kinematics of whole bone fracture simulations, but similar to contemporary isotropic constitutive models, failure was predicted to initiate at the point of loading in an area of high compressive stress, which differed from the reported experiments where failure is reported in the tension region of the bone.

**FIGURE 2:**
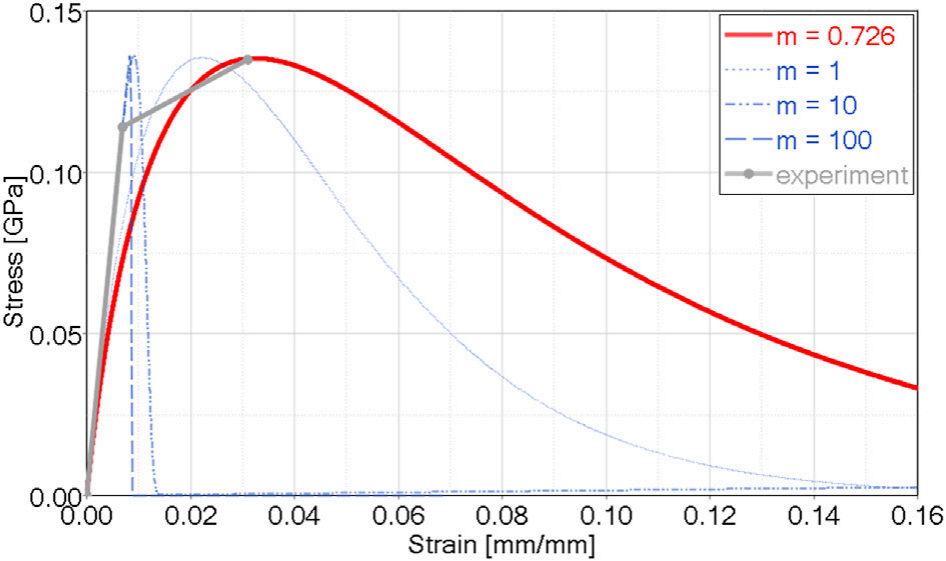
CDM orthotropic and asymmetric model showing damage exponent (m) effect in uniaxial tension, and calibration for cortical bone tensile data (m = 0.726)

CDM models account for damage in a material by reducing the material stiffness to represent microcrack development. The Matzenmiller, Lubliner and Taylor (MLT) post-failure CDM (Matzenmiller et al., 1995; Gower et al., 2007) incorporates orthotropy, asymmetry and material damage. Within the model, damage (*ω*_*ij*_) is represented as a scalar value from 0.0 to 1.0 for each material direction, with increasing damage corresponding to a reduction in material stiffness (*E*_*ii*_ or *G*_*ij*_). Using Voight notation, the rate of damage accumulation,

$$g=\left[\begin{array}{c} \begin{array}{c} e^{-1}\left(1-\omega_{11,t-1}\right)\left(\frac{{\dot{\varepsilon }}_{11}}{\varepsilon_{11,f}}\right){\left(\frac{\varepsilon_{11}}{\varepsilon_{11,f}}\right)}^{m_{11}-1}\\ e^{-1}\left(1-\omega_{22,t-1}\right)\left(\frac{{\dot{\varepsilon }}_{22}}{\varepsilon_{22,f}}\right){\left(\frac{\varepsilon_{22}}{\varepsilon_{22,f}}\right)}^{m_{22}-1}\\ e^{-1}\left(1-\omega_{33,t-1}\right)\left(\frac{{\dot{\varepsilon }}_{33}}{\varepsilon_{33,f}}\right){\left(\frac{\varepsilon_{33}}{\varepsilon_{33,f}}\right)}^{m_{33}-1} \end{array}\\ \begin{array}{c} e^{-1}\left(1-\omega_{12,t-1}\right)\left(\frac{{\dot{\varepsilon }}_{12}}{\varepsilon_{12,f}}\right){\left(\frac{\varepsilon_{12}}{\varepsilon_{12,f}}\right)}^{m_{12}-1}\\ e^{-1}\left(1-\omega_{23,t-1}\right)\left(\frac{{\dot{\varepsilon }}_{23}}{\varepsilon_{23,f}}\right){\left(\frac{\varepsilon_{23}}{\varepsilon_{23,f}}\right)}^{m_{23}-1}\\ e^{-1}\left(1-\omega_{31,t-1}\right)\left(\frac{{\dot{\varepsilon }}_{31}}{\varepsilon_{31,f}}\right){\left(\frac{\varepsilon_{31}}{\varepsilon_{31,f}}\right)}^{m_{31}-1} \end{array} \end{array}\right],$$ (1)

is defined by the damage exponent (*m*_*ij*_) (Figure 2), which is fit to coupon-level material test data. Within the damage calculation, the current strain (ε_*ij*_), current strain rate (ε̇_*ij*_), material failure strain (ε_*ij,f*_) defined as the material strength divided by the modulus, and damage parameter for a given material direction are used to determine the onset of damage through a damage initiation criterion. The damage rate multiplied by the time increment (dt) determines the increase in material damage for a given time increment,

$$\omega_{t}=\omega_{t-1}+g_{t}\;dt,$$ (2)

and is applied in the material compliance tensor,

$$H=\left[\begin{array}{cccccc} \frac{1}{\left(1-\omega_{11}\right)E_{11}} & \frac{-\nu_{21}}{E_{22}} & \frac{-\nu_{31}}{E_{33}} & 0 & 0 & 0\\ \frac{-\nu_{12}}{E_{11}} & \frac{1}{\left(1-\omega_{22}\right)E_{22}} & \frac{-\nu_{32}}{E_{33}} & 0 & 0 & 0\\ \frac{-\nu_{13}}{E_{11}} & \frac{-\nu_{23}}{E_{22}} & \frac{1}{\left(1-\omega_{33}\right)E_{33}} & 0 & 0 & 0\\ 0 & 0 & 0 & \frac{1}{\left(1-\omega_{12}\right)G_{12}} & 0 & 0\\ 0 & 0 & 0 & 0 & \frac{1}{\left(1-\omega_{23}\right)G_{23}} & 0\\ 0 & 0 & 0 & 0 & 0 & \frac{1}{\left(1-\omega_{31}\right)G_{31}} \end{array}\right]$$ (3)

Damage coupling between directions is included, and is visible in the inverted compliance (stiffness) form of the matrix. Importantly, the CDM approach can distinguish between tension and compression and therefore include material asymmetry. The stresses corresponding to the damaged material state are calculated considering the material damage,

$$\sigma =H^{-1}\varepsilon .$$ (4)

Fracture initiation and propagation in bone has been investigated using cohesive-zone element models (Ural & Vashishth, 2006; Ural et al., 2011) with the limitation that the crack-extension path must be predefined (Li et al., 2013). Although the extended finite element method (X-FEM) allows for crack propagation to be modelled without the need to predefine the crack path (Li et al., 2013), current cortical bone models utilizing the X-FEM method are only two-dimensional and are often applied at the micro-scale level (Budyn et al., 2008; Feerick et al., 2013; Abdel-Wahab et al., 2012; Idkaidek & Jasiuk, 2017) prohibiting general use in HBMs.

It has been demonstrated that hydrostatic stress state plays a role in the failure of low ductility materials (Johnson & Holmquist, 1992; Morin et al., 2010). Recent developments in modeling fracture have identified the dependence of failure parameters on stress triaxiality (Butcher & Abedini 2019) for fracture of metals. Triaxiality (η) is defined as the ratio of hydrostatic stress to effective stress, such that a triaxiality of zero corresponds to pure shear loading and a value of 1/3 corresponds to uniaxial tension. The element deletion method (element erosion) with a strain-based failure criterion is still widely used to predict material fracture, and has been somewhat successful at predicting the onset of failure (DeWit, & Cronin, 2012; Schileo et al., 2008; Niebur et al., 2000). The element erosion method is often numerically stable, but has generally been unable to predict bone fracture patterns for various modes of loading.

### 1.2 Experimental testing of whole bones

Whole bone testing is considered to be the most relevant representation of bone response and failure (Shore et al., 2013) since there are no specimen machining artifacts induced. Several studies have been undertaken to measure the mechanical response of whole bones in the lower extremity (Cezayirlioglu et al., 1985; Kress et al., 1995; Strømsøe et al., 1995; Cristofolini et al., 1996; Funk et al., 2004). Khor et al. (2018) identified two whole bone femur load cases to assess constitutive models: a three-point bending load case, including both posterior-anterior and medial-lateral loading conditions (Funk et al., 2004; Kerrigan et al., 2003), and an axial torsion load case (Martens et al., 1980), using an apparatus designed by Burstein and Frankel (1971). The experimental results (Table 2) demonstrated expected fracture patterns for torsional and bending loading.

**TABLE 2:** Whole bone femur experimental data.

		3 point bending	Torsion
Study		(Funk et al., 2004)	(Martens et al., 1980)
Number of Specimens		7	47
Failure Force \ Torque	Average (standard deviation)	4293.6 (560.3) N	183 (54) Nm
	Maximum	4943 N	286 Nm
	Minimum	3646 N	111 Nm
Failure Displacement \ Rotation	Average (standard deviation)	16.7 (3.38) mm	20 (4.5)°
	Maximum	23.3 mm	30.7°
	Minimum	13 mm	9.4°
Fracture Pattern		Tension/wedge Oblique	Spiral Fracture

Initiation of cortical bone fracture is often associated with tensile loading, and locally a defect or stress concentration on the bone surface determines the fracture onset location (Carter & Spengler, 1982). Following initiation, the fracture propagates approximately perpendicular to the maximum principal stress. *In vivo* fractures often exhibit complex fracture patterns due to complex loading (Carter & Spengler, 1982) and higher loading rates tend to increase comminution or fragmentation of the fracture (Kress et al., 1995; Turner, 2006). However, single mode loading fracture patterns are well-established. The strength of cortical bone is lowest in tension and shear, compared to compression (Turner, 2006) and therefore cracks tend to propagate along tension or shear planes within the bone tissue. The fracture pattern associated with tensile loading is a transverse fracture oriented perpendicular to the longitudinal or osteon direction. In bending, failure initiates on the tensile side of the bone due to the lower strength in tension compared to compression (Kress et al., 1995; Turner, 2006; Sharir et al., 2008). As the crack propagates transversely across the bone towards the compressive stress zone, the fracture often bifurcates at an angle of approximately 45° (Turner, 2006; Sharir et al., 2008). This is known as a butterfly or tension wedge fracture and is a typical fracture pattern for bending loading (Sharir et al., 2008). Torsion loading results in a spiral fracture pattern, explained by the maximum tension plane located at 45° from the shear plane, causing the crack to propagate along a helical plane of maximum tension (Turner, 2006).

## 2 Methods

The present study investigated cortical bone response using three models: a contemporary metals plasticity model (Khor et al., 2018), a CDM model using measured mechanical properties of cortical bone with fracture occurring based on damage accumulation, and the CDM model including a novel phenomenological fracture criterion. Bone fracture patterns were assessed using three test cases: notched shear (Iosipescu) specimen, whole femur three-point bending, and whole femur axial torsion. The models were solved in a commercial explicit FE program (LS-DYNA R9.3, LST, Livermore, CA) compiled with a custom code (user material model) for the CDM model and fracture criterion, described below.

### 2.1 Iosipescu (notched shear) specimen simulations

**FIGURE 3:**
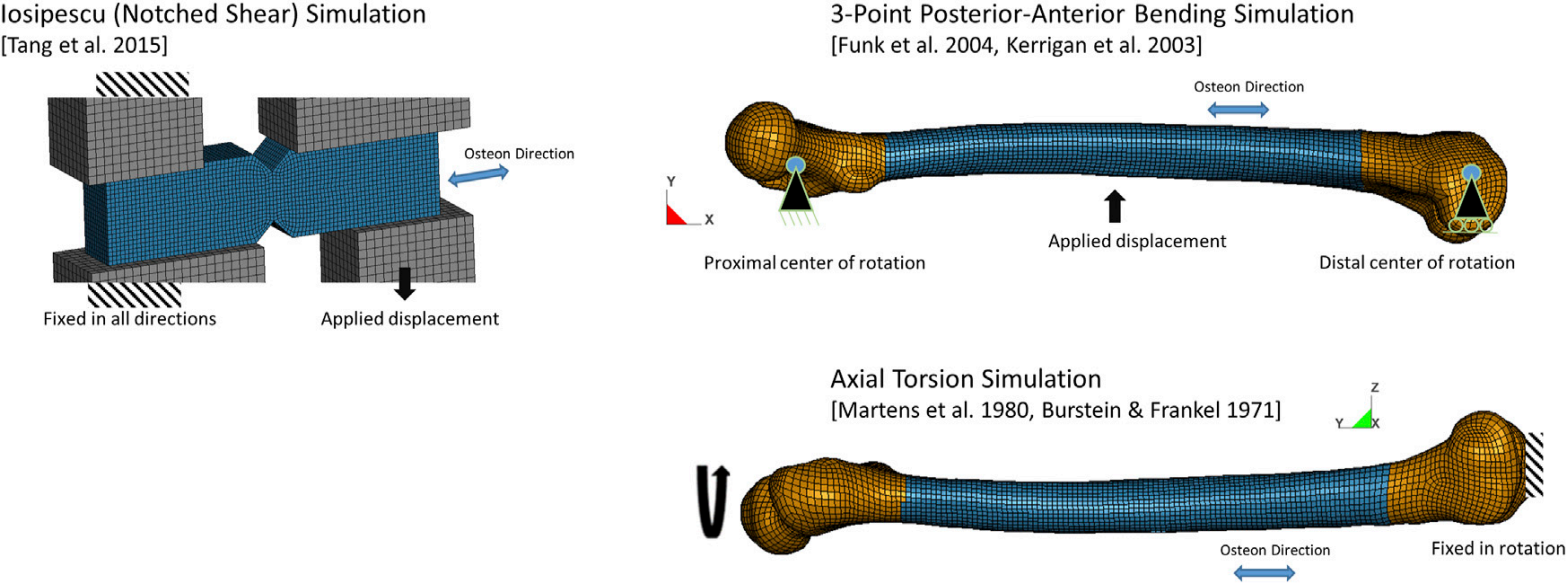
Finite element models of load cases and boundary conditions.

Models of the notched shear test specimen (Figure 3, blue) and loading apparatus (grey) were created in a commercial preprocessor software (Hypermesh, Altair) using the specimen dimensions and boundary conditions described in the experiments (Tang et al., 2015). The loading apparatus was modeled with an elastic material with the properties of steel, and loading was applied at a rate of 0.01 m/s using a displacement boundary condition as in the experiments. The notched shear simulation was used initially to investigate failure criteria and to develop the proposed failure criterion. The model response was verified by comparing to the experimental force-displacement response.

### 2.2 Whole femur finite element model

The femur finite element model was extracted from a current average stature (50th percentile) male human body model (M50 Version 6.0, Global Human Body Models Consortium). In a previous study, this femur model was shown to fall within one standard deviation in length and cross-sectional area reported in the experiments (Khor et al., 2018). Owing to the continuum nature of the constitutive model in this study, the osteons were not explicitly modeled; however, the effect of the osteon orientation and connectivity is known to be associated with fracture patterns and was included in the model by assigning the nodes of each element to define element-level material directions (Supplementary Figure S1). The primary material direction was the osteon direction, and the two orthogonal directions were radial (through thickness) and circumferential, as required for the orthotropic CDM model.

#### 2.2.1 Three-Point bending whole femur load case

The three-point bending boundary conditions were applied to the femur at the epiphyses (Figure 3). The proximal femur translation in the model was fixed in the inferior-superior, anterior-posterior, and medial-lateral directions (*x*, *y* and *z*-directions, respectively, in the model). The distal femur translation was fixed in the anterior-posterior, and medial-lateral directions (*y* and *z*-directions, respectively, in the model). Both epiphyses were free to rotate about the direction perpendicular to the loading, as described in the experimental studies. The long axis of the bone was aligned with the global x-axis of the model and loading was applied by a steel semi-cylinder near the midpoint along the diaphysis, corresponding to the loading point in the experimental tests. The semi-cylinder was moved (1.2 m/s) using a prescribed displacement condition and load was monitored through the contact force. The contact algorithm between the semi-cylinder and the bone accounted for element erosion, and reformulated the contact surface after each eroded element was removed to represent the loading and interaction with the bone as fracture progressed. The model response was validated by comparing to the experimental force-displacement response.

#### 2.2.2 Axial Torsion whole femur load case

The femur was aligned with the global *y*-direction for the axial torsion load case (Figure 3) with the distal epiphysis of the bone fixed in rotation and translation, and the proximal epiphysis loaded using a prescribed rotation displacement (0.00873 rad/ms or 500^°^/s). The model response was validated by comparing to the experimental torque-rotation response.

### 2.3 Orthotropic continuum damage mechanics model and triaxiality-based failure criterion

Within the current study, three material models were analyzed. The reference case was an isotropic plasticity model as implemented in contemporary HBMs, with failure predicted by element erosion at an effective plastic strain of 1.8% (Khor et al., 2018). The second case was the MLT CDM, implemented with element failure occurring at a damage value of 1.0. The MLT implementation enabled for the assessment of material orthotropy, tension-compression asymmetry and damage. Thirdly, a novel stress triaxiality failure criterion was integrated with the MLT CDM model, described as the Cortical Bone Fracture and Continuum Damage Mechanics Model (CFraC).

#### 2.3.1 MLT continuum damage mechanics model

The MLT CDM model was implemented as a user-defined material model as reported in the literature (Gower et al., 2007), in a commercial explicit FE program. The model enabled simulation of the elastic and the CDM portions of the tensile response (Figure 1) using published material properties. The material model parameters were determined as follows:

1) Elastic moduli, Poisson’s ratios and strength values were taken from the literature (Table 1). Although cortical bone properties do exhibit variability, the average elastic mechanical properties and strength properties are generally agreed upon in the literature (Khor et al., 2018).
2) The tensile damage exponent in the osteon direction (1-direction) (*m*_*11*_) and transverse directions (*m*_*22*_, *m*_*33*_) were determined using single element simulations of uniaxial tensile test data in the respective directions, with equivalent strain energy density in the model and experiment (i.e. the same area under the stress-strain curve) (Figure 2; Supplementary Figure S2). In general, the shape of the MLT CDM model differed from the reported experimental data, in that the experimental data demonstrated a more abrupt change from elastic to CDM regions, with a shallow slope in the CDM region. This difference is a limitation of the MLT model formulation and should be investigated for future CDM approaches.
3) It was assumed that damage and failure at the element level did not occur directly in compression or shear (i.e., ω_12_, ω_23_ and ω_31_ were zero in Eq. 3). However, failure of an element could occur in these modes of loading due to the implemented damage coupling with other material directions.

#### 2.3.2 Stress triaxiality-based failure criterion (CFraC)

The observation that hydrostatic stress plays a role in the failure of low ductility materials, and the dependency of failure on stress triaxiality led to the investigation of a novel criterion to represent the fracture mechanics (failure) region of cortical bone (Figure 1A) in the present study. It was hypothesized that damage accumulation and localization may be a function of stress triaxiality and effective strain. This hypothesis, which still requires experimental verification, was pursued in the current study. The proposed fracture criterion was coupled to the MLT CDM model to create the Cortical Bone Fracture and Continuum Damage Mechanics Model (CFraC) and investigated using three test cases (Iosipescu test, three-point bending, axial torsion). A fourth case (uniaxial tension) was introduced with a known tensile hydrostatic stress (1/3 of the material strength in a given direction) and stress triaxiality (1/3). The effective strain *versus* triaxiality curve was determined by simulating each of the three load cases (Iosipescu test, three-point bending, axial torsion) without failure. At the force or torque corresponding to failure in the experiments, the effective strain and corresponding triaxiality were determined for each load case. Element failure (erosion) was based on the effective strain *versus* triaxiality curve constructed using each of the load cases. The critical hydrostatic stress values were determined from the finite element femur model so that the presented values were relevant to the finite element mesh sizes used in contemporary HBMs. However, finite element models and simulation of failure processes are known to have a dependence on finite element mesh size. In the present study, these effects were investigated using two refined models with mesh densities increased by a factor of 2x and 4x. The results were compared between different mesh sizes to assess the effect of mesh refinement on the predicted mechanical response and failure.

To verify the material model implementation in the FE code, single element simulations were undertaken for the osteon and transverse directions in tension, compression and shear. A mesh size of 3 mm was used for these simulations. In each case, the element was fixed in the loading direction with one-eighth symmetry conditions applied, and displacement boundary conditions were applied to the opposite element face. The element was not constrained opposite the symmetry planes, allowing for deformation due to Poisson’s ratio effects. Implementing the orthotropic material properties (Table 1) in the MLT model, single element simulations were undertaken in longitudinal (osteon direction) tension/compression, orthogonal (circumferential and radial directions) tension/compression, and in-plane shear direction. All models provided the expected stress-strain response, which verified the model implementation and input data.

## 3 Results and discussion

### 3.1 Failure criterion: Effective strain versus stress triaxiality

**FIGURE 4:**
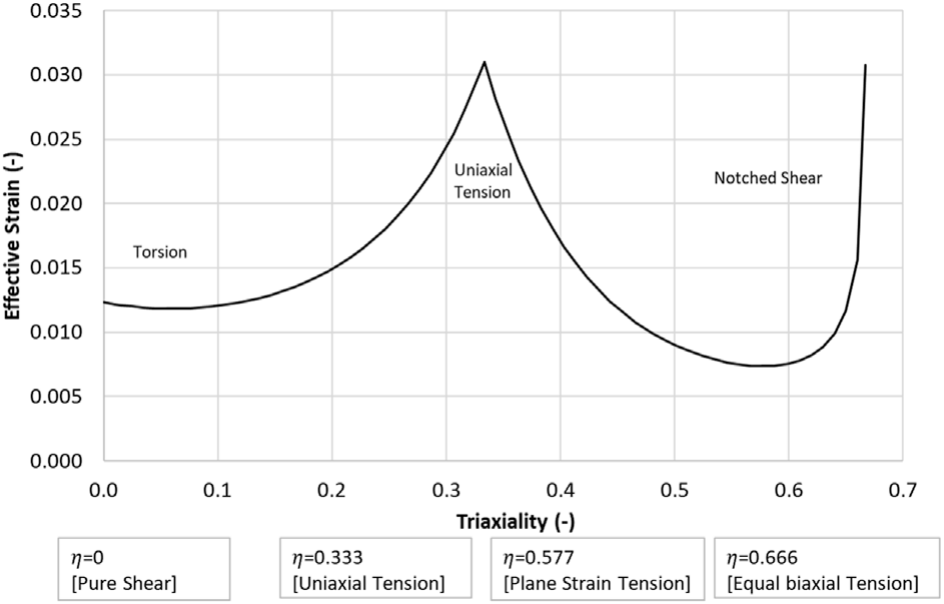
Effective strain *versus* stress triaxiality (η) failure criterion.

The fracture initiation threshold curve, describing hydrostatic stress as a function of triaxiality (*η*), was plotted for the three load cases (data points, Figure 4 and Table 3), along with the reported uniaxial tensile failure value at a triaxiality of 1/3. The data were then fit to a Modified Mohr-Coulomb model (Kõrgesaar et al., 2018), which was implemented in the computational model to define the effective strain at failure (*ε̅*_*f*_) *versus* triaxiality:

$${\bar{\varepsilon }}_{f}={\left\{\frac{K_{m}}{C_{2}}f_{3}\left[\sqrt{\frac{1+C_{1}^{2}}{3}}f_{1}+C_{1}\left(\eta +\frac{f_{2}}{3}\right)\right]\right\}}^{-1/n_{m}}$$, (5a)

$$f_{1}=\cos\left\{\frac{1}{3}\arcsin\left[-\frac{27}{2}\eta \left(\eta^{2}-\frac{1}{3}\right)\right]\right\},$$ (5b)

$$f_{2}=\sin\left\{\frac{1}{3}\arcsin\left[-\frac{27}{2}\eta \left(\eta^{2}-\frac{1}{3}\right)\right]\right\},$$ (5c)

$$f_{3}=C_{3}+\frac{\sqrt{3}}{2-\sqrt{3}}\left(1-C_{3}\right)+\left(\frac{1}{f_{1}}-1\right).$$ (5d)

**TABLE 3:** Modified Mohr-Coulomb fracture model parameters.

MMC model parameter	Value
C_1_	0.04415
C_2_	1.0
C_3_	0.00210
K_m_	0.38703
n_m_	0.08347

Although the proposed failure locus is empirical at present, it highlights potential need for additional experimental information considering varying triaxiality, as was achieved in the Iosipescu samples (Tang et al., 2015) with high triaxiality at the failure location adjacent to the notch.

### 3.2 Iosipescu notched shear simulation

**FIGURE 5:**
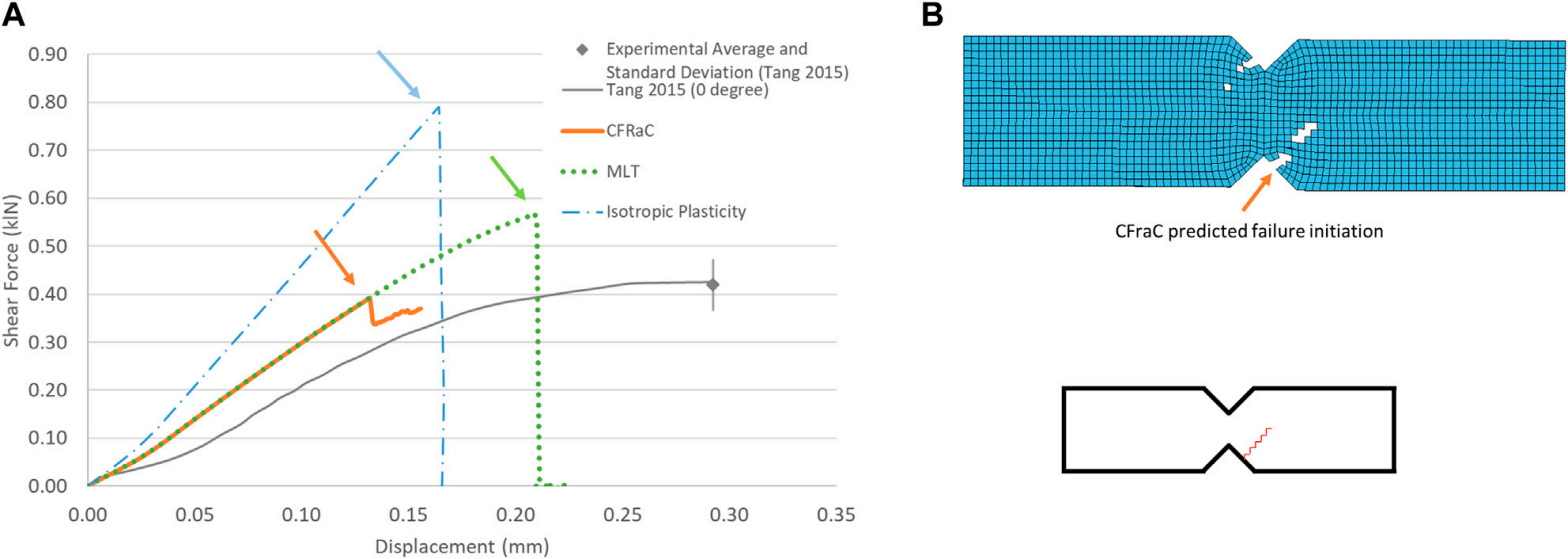
Force-displacement response for notched shear (Iosipescu) specimen (experimental response from Tang et al., (2015) **(A)**; and predicted fracture *via* eroded elements **(B)**, fracture initiation location indicated by arrow.

The notched shear test simulation demonstrated a monotonically increasing force *versus* displacement response up to the initiation of failure (Figure 5). The isotropic plasticity model significantly over predicted the failure force in shear due to the yield surface assumption in the model, highlighting a significant limitation of plasticity approaches to modeling cortical bone under shear loading.

Although the MLT CDM model predicted the failure force (0.5 kN) in reasonable agreement with the experiments, owing to input of the experimentally reported shear strength, failure was predicted in the central, shear gauge area between the two notches of the specimen, which disagreed with the experimental data (Supplementary Figure S3).

The CFraC model predicted a failure force (−0.41 kN), in agreement with the average reported experimental value (−0.42 kN). The simulation exhibited a similar stiffness to the experiment, but did not incorporate a toe region, attributed to compliance in the test fixture and the challenge of experimentally measuring very small displacements. The Iosipescu test specimen demonstrated high triaxiality (*η* = 0.54) at the fracture initiation location, approaching equibiaxial tension (*η* = 0.666). Fracture was predicted to initiate adjacent to the root of the sample notch and propagated at an approximate angle of approximately 40° to the horizontal plane, in agreement with the experimental findings.

### 3.3 Whole femur three-point bending simulation

**FIGURE 6:**
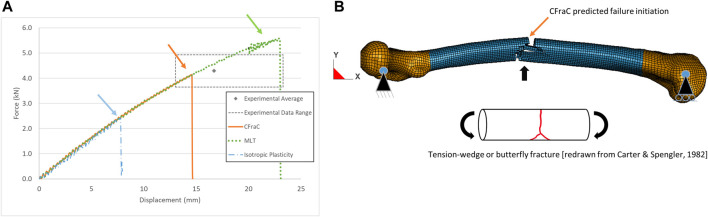
Force-displacement response for whole bone posterior-anterior bending test (experimental response from Funk et al., (2004) **(A)**; and predicted tension-wedge fracture *via* eroded elements **(B)**, fracture initiation location indicated by arrow.

For the whole femur three-point bending load case, the model force-displacement response monotonically increased up to the initiation of failure (Figure 6). The isotropic plasticity model failure force was below the test average and range owing to failure initiation at the load point early in the load history. Progressive crushing of the bone at the load point occurred providing an apparent energy absorption, but the fracture pattern did not agree with those reported in the literature.

The MLT model predicted a failure force above to the experimental average. Although the fracture initiated on the tension side of the bone, the fracture bifurcated to follow the neutral axis of the bone (Supplementary Figure S3) due to the lower shear strength of the bone, and was not representative of reported fracture patterns.

The maximum force of the CFraC model in posterior-anterior bending (4.15 kN) was within the range of the experimental data (3.6–4.9 kN). The displacement at failure (14.6 mm) agreed with the experimental average (16.7 mm, standard deviation of 3.38 mm). In medial-lateral bending, the maximum force was 3.5 kN at a displacement of 15.8 mm. Additional simulations demonstrated that achieving a higher force value required an increase in the tensile material strength (Table 1) or decreasing the damage exponent (m11). The tensile strength of the bones tested in the experiments conducted by Funk et al. (2004) may have differed from properties reported by Reilly et al. (1974) that were used in the constitutive model. The geometry of the femur used in the present study (length and cross-sectional area) was similar to the average reported in the experiments (Khor et al., 2018). However, variations within the population are expected to result in a range of physical dimensions and material properties, which may explain some of the variability in the experimental test results (Table 2). The nonlinear response of the MLT and CFraC models was due to the accumulation of damage and corresponding reduction in stiffness of the cortical bone material. The CFraC model predicted failure initiation on the tension surface of the bone and fracture propagated across the bone to the compression side, as reported in the literature.

### 3.4 Whole femur axial torsion simulation

**FIGURE 7:**
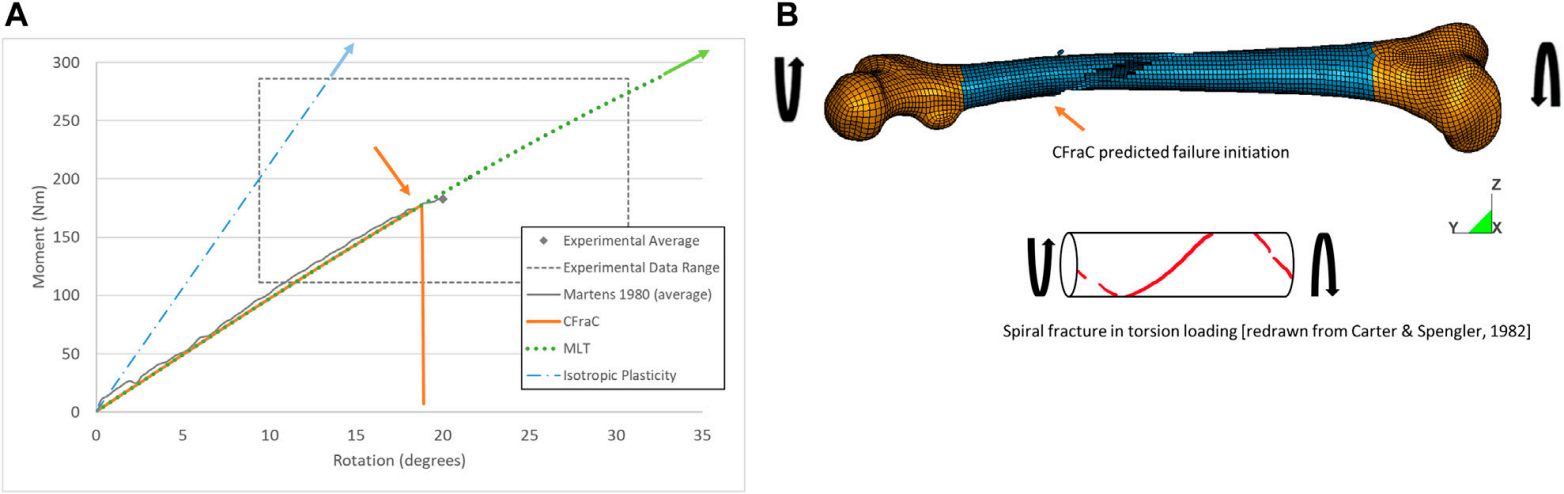
Torque-rotation response for whole bone axial torsion test (experimental response from Martens et al., (1980) **(A)**; and predicted spiral fracture *via* eroded elements **(B)**, fracture initiation location indicated by orange arrow.

When the femur was loaded in axial torsion, the moment rotation response was monotonically increasing and relatively linear up to the point of failure. The isotropic plasticity model predicted a very high torque at failure for the whole femur axial torsion load case (Figure 7), attributed to the yield surface definition in the material model. The MLT model also predicted a torque and axial rotation exceeding the reported experimental range with a linear fracture pattern along the length of the bone (Supplementary Figure S3), rather than a spiral fracture, owing to the low shear strength along the length of the bone.

For the CFraC model, failure initiated at a torque of 182 Nm in the proximal end of the diaphysis, and propagated in a spiral fracture towards the distal end of the bone. The predicted rotation to failure (19°) was within the experimental bounds (9.4–30.7°) and comparable to the reported experimental average (20°). The axial rotation for a given axial torque was determined by the torque, polar second moment of area, femur effective length, and shear modulus. Thus, increased length, reduced cross-sectional area or reduced stiffness could affect the rotation but were not investigated in the current study. While triaxiality is zero for pure shear loading, the triaxiality in the diaphysis under axial torsion ranged from zero up to ∼0.1. The varying η value was associated with changes in geometry of the bone. It should be noted that the failure location was within the diaphysis, and away from the epiphyses and applied boundary condition effects. This small but non-zero η value highlights the potential importance of the local bone geometry on response and failure prediction.

### 3.5 CFraC finite element model mesh refinement

A mesh refinement study was undertaken for the CFraC constitutive and fracture model using the three load cases. Finite element models are known to have a dependence on finite element size, attributed in part to the modeling of stress or strain gradients, while modeling of failure may be a non-convergent phenomenon. The finite element meshes were refined by splitting all solid elements (1 element split to 8 elements) and all shell elements (1 element split to 4 elements). This procedure was carried out twice to provide two refined meshes per model, in addition to the baseline mesh. All analyses were run to the same termination time, using the same boundary conditions and material properties. In general, refining the mesh resulted in a slightly lower predicted failure force or moment and failure displacement or rotation (Supplementary Figure S4), due to the improved resolution of the high hydrostatic tension gradient in the failure zone. Each refined model demonstrated the same location of fracture initiation and a more refined fracture pattern. Since the objective of this study was to provide a cortical bone material model for use in contemporary HBM, the critical effective strain was determined from the coarse mesh (production mesh for the GHBMC M50 model).

### 3.6 General discussion

The proposed failure initiation criterion was fit to the experimental data using the simulations so it should be expected that the models would predict the onset of failure in agreement with the experiments. In practice, mechanical tests are undertaken using specimen geometries that achieve varying levels of triaxiality using, for example, tensile test specimens with various notch geometries. The Iosipescu sample presents an example of this type of test. Although the goal of this shear test was to achieve pure shear loading (*η* = 0) at the failure location, this case demonstrated the highest triaxiality of all cases considered. In general, material failure data as a function of triaxiality is not currently available for cortical bone; however, the current model suggest this experimental data would be useful to further refine the predictive capabilities of cortical bone models.

One aspect not considered in the current model are deformation rate effects. Studies have identified deformation rate effects in compression loading (McElhaney, 1966) comprising increased modulus, increased strength and decreased strain to failure with increasing loading rates. However, studies are generally not in agreement regarding deformation rate effects in tension loading, where some researchers have measured increasing (Melnis & Knets, 1982) and reduced (Hansen et al., 2008) stiffness and strength with increasing loading rates. Given that tension appears to be an important factor in accumulating damage, and experimental studies to date present inconsistent information, deformation rate effects were not included in the current study. Further mechanical testing is needed to better quantify the effect of deformation rate on the mechanical properties of bone. The present study investigated fractures as reported in the literature that were generated from dynamic loading relevant to automotive crash or sports injuries. Additional three-point bending and torsion testing across a range of loading rates would provide some insight into the evolution of the facture pattern with loading rate. This potential evolution could then be considered in the model by implementing a strain-rate dependent fracture criterion. It is important to note that very high rate loading such as that encountered in blast exposure or ballistic impact may result in less well-defined fracture patterns, and more comminuted fracture. For example, exposure to antipersonnel landmines often results in comminuted fractures of foot and long bones (Cronin et al., 2011) while ballistic impacts on bone may result in drill-hole type fractures in the ribs and sternum (Nguyen et al., 2022).

The proposed model was symmetric in shear (in-plane direction) while the bone structure is asymmetric in the two in-plane shear directions owing to the orientation of the osteons and cement lines. Therefore, only shear across the osteons or parallel to the osteons can be represented. Within the current model, shear across the osteons was incorporated into the model, and provided good predictions of fracture strength and pattern for the axial torsion and Iosipescu cases. Material data in shear is somewhat limited, owing to the challenges in experimentally achieving pure shear loading, and the complex failure modes exhibited by cortical bone. The study by Tang et al. (2015) demonstrated that shear loading of cortical bone transverse to the osteon direction leads to complex failure behavior, although the measured force-displacement response is believed to represent the material shear behavior up to the point of fracture initiation. The Iosipescu sample should have zero triaxiality through the intended gauge section, but actually demonstrates very high triaxiality at the failure location owing to the presence of the notch. Current work in fracture of materials uses various notched samples in tension and shear to measure material failure for different levels of *η*. Such testing should be undertaken for bone in the future. The present study provides an estimate of the failure curve by interpreting existing experiments, but was limited in terms of material data available for a range of *η* values.

In the models of the Iosipescu tests, the displacement to failure was under predicted compared the experiments. This may be related to compliance in the experimental test apparatus, and variations between the moduli used in the simulations compared to the tests.

The constitutive and fracture model was computationally stable in all cases, allowing for the prediction of loading, fracture, and post-fracture response of the bone. Importantly, the CFraC model was able to predict the ultimate load, initiation of fracture and fracture pattern for the common modes of loading. The computation time for the whole femur subjected to axial torsion was 1.59 times greater for the CFraC model (Supplementary Figure S5) compared to the isotropic plasticity model, when simulated on one compute core of a Symmetric Multiprocessing system (i9 9960x CPU at 3.1 GHz). This ratio decreased to 1.02 when using 16 cores. In general, the CFraC model was more computationally expensive than the isotropic plasticity, which is anticipated owing to the larger number of calculations required for the CFraC model.

## 4 Study conclusions and limitations

The isotropic material model used in contemporary HBMs was able to predict the failure force associated with tension-based failures (e.g. three-point bending) but significantly over predicted the failure force or torque for shear loading due to the yield surface assumption embedded in metals plasticity models. An orthotropic MLT CDM material model including tension-compression asymmetry provided improved kinetic predictions for the load cases considered but was not able to predict fracture patterns in agreement with reported observations.

The proposed fracture initiation criterion (CFraC) was phenomenological, based on available experimental data and calibrated to the whole bone tests with respect to the failure initiation threshold; however, the resulting fracture patterns were not calibrated in any way. Importantly, the proposed failure criterion enabled the prediction of cortical bone fracture patterns in agreement with experimentally observed data. Refining the finite element mesh resulted in reduced force or moment and displacement to failure, as expected; and led to more refined fracture patterns that were in good agreement with reported bone fracture patterns for different modes of loading.

The study employed a subject-specific femur bone model, with length and cross-sectional area similar to the average values presented in experimental studies, and therefore was expected to be representative for the purposes of the current study. Variability in material properties and femur geometry were not investigated in the current study but may explain some of the differences between the model predictions and the average experimental results.

The CFraC constitutive and fracture model was computationally stable in all three load cases, allowing for the prediction of loading, fracture, and post-fracture response of the bone. Importantly, the model was able to predict the ultimate load, initiation of fracture and fracture pattern for the common modes of loading.

## Data Availability

The novel material model developed in this study will be available to the research community as a shared library that can be linked into the LS-DYNA finite element code. Please contact the corresponding author for information on accessing the material model.

## Nomenclature

CDM: Continuum Damage Mechanics
CII: Crash Induced Injuries
FE: Finite Element
HBMs: Human Body Models
MLT: Matzenmiller, Lubliner and Taylor
X-FEM: Extended Finite Element Method
C_1_, C_2_, C_3_, K_m_, n_m_: Modified Mohr Coulomb Model fracture parameters
*E*_*i*_: Young’s modulus in *i* direction
*G*_*ij*_: Shear modulus in *ij* plane
*K*: Bulk Modulus
*S_i_*: Ultimate Strength in i direction
*S_ij_*: Ultimate Strength in ij plane
*ν_ij_*: Poisson’s ratio in ij plane
*m_ij_*: MLT constitutive model damage rate exponent
*ω_ij_*: Damage value associated with a specific material direction
*g_ij_*: Damage rate corresponding to wij
*η*: Triaxiality
*ε_ij_*: Current strain
*ε̇_ij_*: Current strain rate
*ε_ij,f_*: Material failure strain
*ε̅_f_*: Effective strain at failure
