# Supplementary material for: Cortical bone continuum damage mechanics constitutive model with stress triaxiality criterion to predict fracture initiation and pattern
Source: Front Bioeng Biotechnol. 2022 Oct 17;10:1022506. doi: 10.3389/fbioe.2022.1022506 (PMC9618659; doi:10.3389/fbioe.2022.1022506)
Supplement: Supplementary file 1 [file DataSheet1.docx]

# Appendix A: Supplemental Figures

Figure A1: Whole femur finite element mesh and material directions

Figure A2: MLT continuum damage mechanics model, calibration of damage exponent (m)


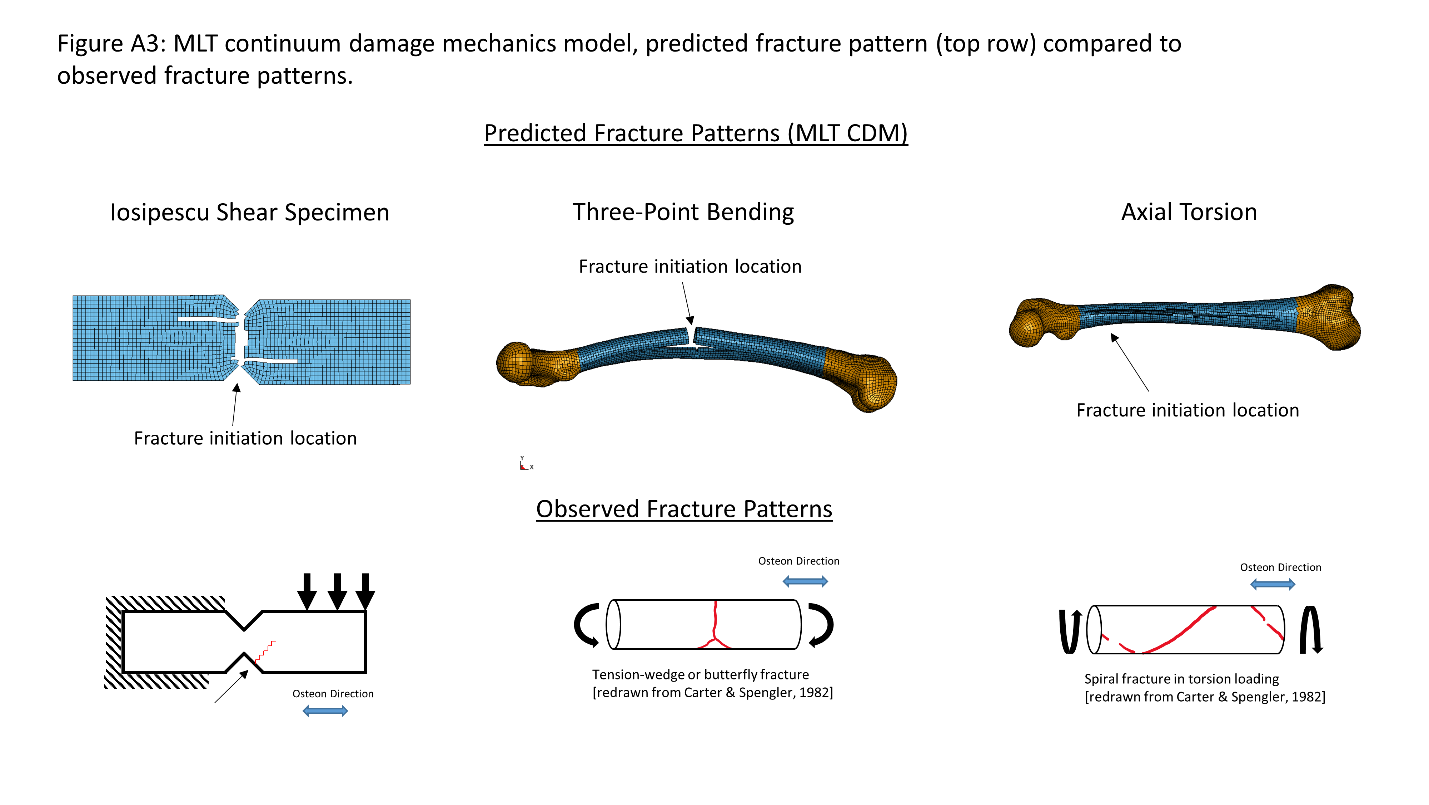


Figure A3: MLT continuum damage mechanics model, predicted fracture pattern (top row) compared to observed fracture patterns


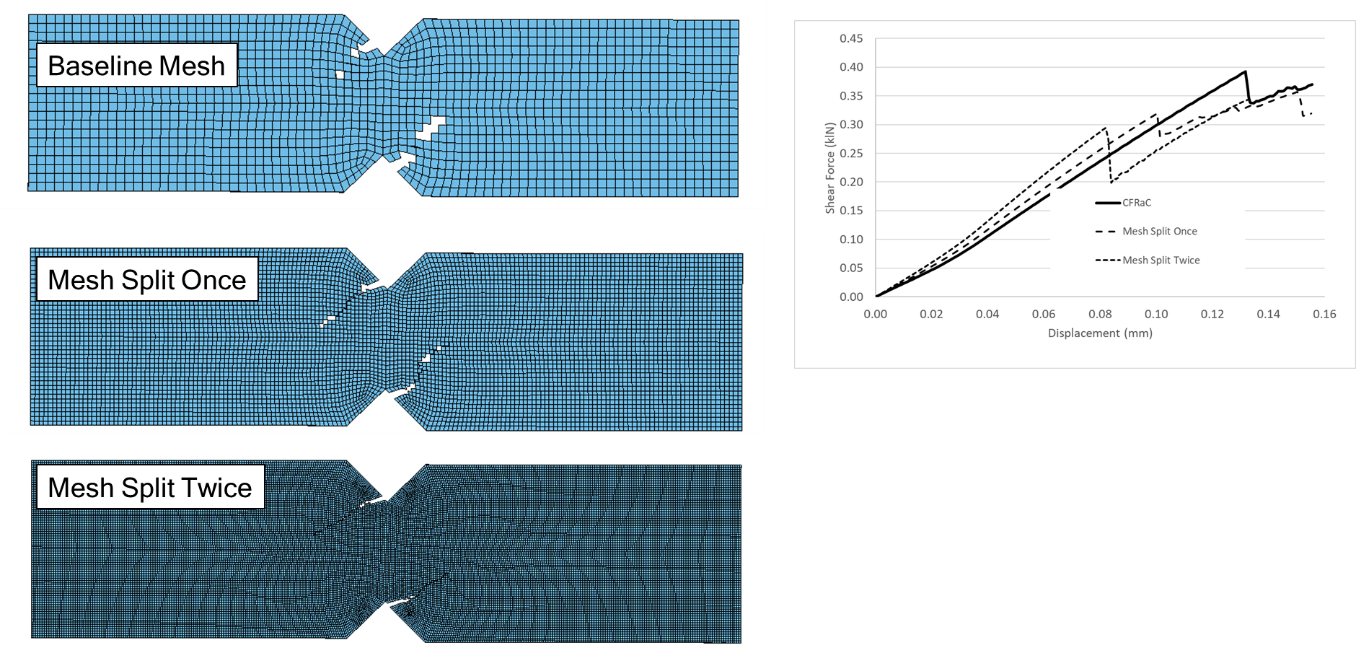


Figure A4: Notched shear specimen and effect of finite element mesh refinement for CFraC model


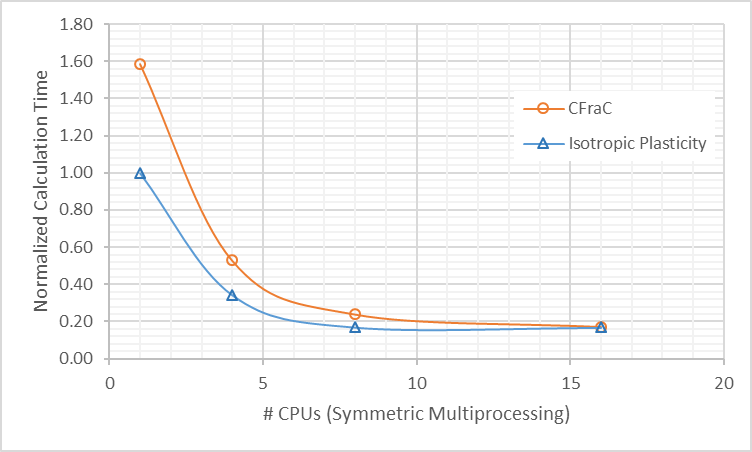


**Figure A5: Normalized compute time of the CFraC model, compared to the isotropic plasticity model for the femur model subjected to axial torsion**
